# Cardiac arrest as the initial presentation of thyrotoxicosis in a young woman

**DOI:** 10.1530/EDM-25-0176

**Published:** 2026-04-13

**Authors:** Maria Helena Siqueira Tavares de Melo, Maxim John Levy Barnett, Maria L M Rego, Evan Isaacs, Elilta Desta, Ana Rivadeneira

**Affiliations:** Jefferson Einstein Philadelphia Hospital, Philadelphia, Pennsylvania, USA

**Keywords:** thyroid storm, cardiac arrest, vasospasm, thyrotoxicosis, thyroid

## Abstract

**Summary:**

Thyroid storm is a life-threatening endocrine emergency characterized by severe thyrotoxicosis and multisystem decompensation. Cardiovascular involvement is common and the leading cause of mortality, most commonly presenting with atrial fibrillation or high-output heart failure. Malignant ventricular arrhythmias, however, are exceedingly rare, reported in 0.07% of hospitalizations for thyroid dysfunction and 13% of patients with thyroid storm admitted to intensive care units. We present a case of a previously healthy young woman with uncontrolled Graves’ disease who developed out-of-hospital cardiac arrest due to ventricular fibrillation. Coronary angiography revealed diffuse coronary vasospasm without obstructive coronary artery disease, suggesting myocardial ischemia as the precipitating mechanism. She achieved complete neurological and hemodynamic recovery following resuscitation, initiation of antithyroid therapy, beta-blockade, corticosteroids, and restoration of euthyroidism. This case underscores the potential of thyroid storm to induce life-threatening ventricular arrhythmias through a combination of coronary vasospasm, sympathetic overactivity, and altered myocardial excitability. Recognition of thyrotoxicosis as a potential cause of cardiac arrest is crucial, particularly in patients without structural heart disease, since early diagnosis and timely treatment are key to survival and prevention of recurrence.

**Learning points:**

- Ventricular fibrillation and cardiac arrest, even in patients without structural heart disease, can be a presentation of thyroid storm.
- Coronary vasospasm represents a potential mechanism linking thyrotoxicosis to malignant ventricular arrhythmias.
- Prompt recognition and restoration of euthyroidism are essential for reversing hemodynamic instability and preventing recurrence.
- Nonadherence to antithyroid therapy remains a major preventable trigger of thyroid storm.

## Background

Thyroid storm is a rare but life-threatening endocrine emergency characterized by multiorgan dysfunction resulting from severely uncontrolled hyperthyroidism. This condition carries a high risk of mortality, with death rates ranging between 3.6 and 17% depending on age, comorbidities, and extent of systemic decompensation (1). It arises from thyrotoxicosis, a state of markedly elevated circulating thyroid hormones most often caused by Graves’ disease, amiodarone-induced thyrotoxicosis, and postpartum thyroiditis, and is commonly precipitated by nonadherence to antithyroid therapy (1, 2).

Thyroid storm is associated with diverse cardiovascular complications, ranging from sinus tachycardia and atrial fibrillation to high-output heart failure and, in rare cases, malignant ventricular arrhythmias or cardiac arrest (3). These cardiovascular complications result from the combined effects of thyroid hormone excess on the autonomic nervous system, vascular endothelium, and myocardial excitability. Thyroid hormones enhance β-adrenergic receptor sensitivity, increase calcium handling in vascular smooth muscle and cardiac myocytes, and impair nitric oxide-mediated vasodilation, collectively promoting sympathetic overactivity, vasospasm, and arrhythmogenesis (4, 5, 6).

The diagnosis is primarily clinical, based on severe thyrotoxicosis with evidence of systemic decompensation. Scoring systems can aid in assessment, with a Burch–Wartofsky Point Scale ≥ 45 or Japanese Thyroid Association categories TS1/TS2 indicating the need for aggressive therapy (7).

Management requires prompt recognition, intensive care unit (ICU)-level monitoring, and rapid reduction of circulating triiodothyronine (T3) levels. Key therapies include antithyroid drugs, beta-blockers, glucocorticoids, and inorganic iodide to inhibit hormone synthesis and block peripheral conversion of thyroxine (T4) to T3 (8). Propylthiouracil (PTU), historically recommended as first-line therapy particularly in patients with cardiovascular compromise, inhibits thyroid peroxidase and uniquely blocks T4-to-T3 conversion (7). However, recent multicenter U.S. and Japanese registry data demonstrate no significant differences between PTU and methimazole (MMI) in mortality, need for organ support, adverse events, or cost (9).

## Case presentation

A 35-year-old female with a history of Graves’ disease, hypertension, and depression was brought to the emergency department in cardiac arrest. Outpatient prescriptions included methimazole and propranolol, but adherence was uncertain. At presentation, she was not receiving any psychotropic medications or other agents associated with QT interval prolongation. She had lost approximately 27 kg (60 lb) over the past year following her diagnosis of Graves’ disease. The night before the presentation, she experienced chest pain radiating to the left arm, which worsened the following day. While en route to the hospital, she developed seizures and collapsed. EMS found her pulseless in ventricular fibrillation (VF). Upon arrival at the hospital, return of spontaneous circulation was achieved after prolonged resuscitation, although she required a second episode of CPR for recurrent VF. She was intubated, sedated, and transferred to the intensive care unit.

## Investigation

**Figure 1:**
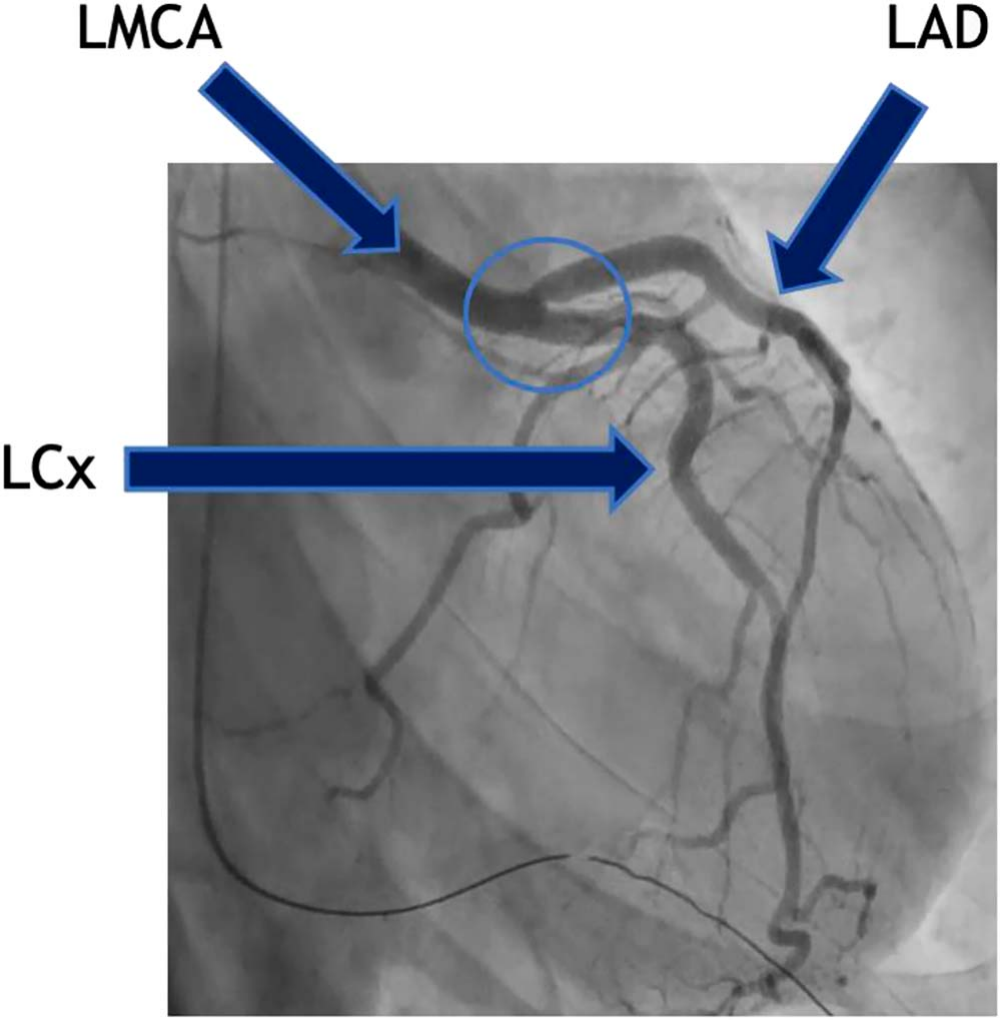
Coronary angiography demonstrating LAD ostial spasm. Right anterior oblique (RAO) caudal angiographic view showing the left anterior descending (LAD) artery coursing across the upper portion of the image. A focal narrowing is seen at the LAD ostium, consistent with coronary vasospasm.

**Figure 2:**
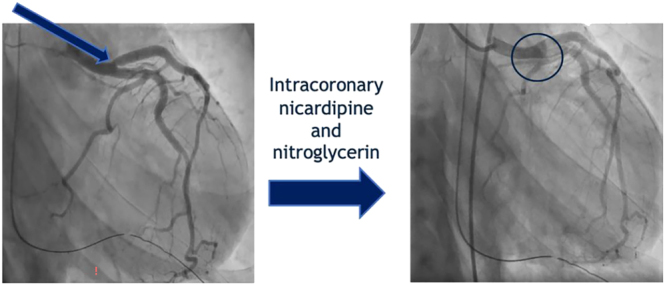
Resolution of LAD ostial spasm following intracoronary vasodilators. Right anterior oblique (RAO) caudal angiographic view obtained after administration of intracoronary nitroglycerin and nicardipine, demonstrating complete resolution of the previously noted LAD ostial narrowing. The vessel lumen appears fully restored, consistent with reversible coronary vasospasm.

Post-cardiac arrest, the patient was admitted to the cardiac intensive care unit. She immediately underwent cardiac catheterization, which revealed angiographically normal coronary arteries with right-dominant circulation. However, vasospasm was observed at the ostia of the left anterior descending, resolving after administration of intracoronary nitroglycerin and nicardipine. (Figs 1 and 2) High-definition intravascular ultrasound confirmed the absence of significant atherosclerotic disease. Hemodynamic assessment demonstrated mild pulmonary hypertension, evidence of left ventricular failure, and a cardiac index of 3.1 L/min/m^2^.

Cardiac biomarkers were abnormal, with troponin rising from 81 ng/L to a peak of 2,292 ng/L and B-type natriuretic peptide measured at 483 pg/mL. Electrocardiogram obtained after cardiac arrest demonstrated sinus tachycardia. Point-of-care ultrasound at admission revealed severely depressed systolic function with an ejection fraction (EF) of approximately 10% and global hypokinesis. A formal transthoracic echocardiogram performed the following day showed interval improvement on cardiac function, with mildly reduced systolic function (EF: 40–45%) and evidence of regional wall motion abnormalities involving the anteroseptal, inferoseptal, inferior, inferolateral, and apical anterior walls.

Initial laboratory evaluation revealed a suppressed thyroid-stimulating hormone (TSH) (<0.01 µU/mL), elevated free T4 (2.33 ng/dL; reference range: 0.82–1.77), and markedly elevated total T3 (435 ng/dL; reference range: 80–200). Her Burch–Wartofsky Point Scale score was 75 (highly suggestive of thyroid storm), and she fulfilled the criteria for a definite case according to the Japan Thyroid Association diagnostic guidelines. The endocrinology team was urgently consulted.

Two days later, repeat testing demonstrated biochemical improvement, with free T4 declining to 1.81 ng/dL and free T3 to 166 ng/dL. Four days after admission, free T4 further decreased to 1.23 ng/dL and total T3 to 92 ng/dL. By the end of the first week, free T4 had normalized (0.96 ng/dL), and total T3 remained within the normal range, although TSH persisted at <0.01 µU/mL.

## Treatment

The patient was managed in the intensive care unit with mechanical ventilation and sedation. Antithyroid therapy was initiated with methimazole 20 mg twice daily, which was subsequently reduced as thyroid hormone levels improved, with the patient discharged on methimazole 15 mg daily. Propranolol was started for adrenergic control at 10 mg twice daily and titrated to 30 mg twice daily to achieve adequate heart rate control. Intravenous hydrocortisone was administered to reduce peripheral conversion of T4 to T3 and to provide adrenal support. Supportive measures included close hemodynamic monitoring and ventilatory management. Before discharge, patient underwent successful placement of an implantable cardioverter defibrillator (ICD).

## Outcome and follow-up

On interval follow-up later during hospitalization, echocardiography demonstrated near normalization of systolic function with an EF of 50%. The exam was unable to assess wall motion abnormality due to poor endocardial resolution.

ICD interrogation upon cardiology follow-up did not demonstrate recurrent ventricular arrhythmias or episodes of supraventricular arrhythmias.

## Discussion

Thyroid storm is strongly associated with major adverse cardiovascular events, the leading contributors to morbidity and mortality (3). In a recent national analysis of 79,831 U.S. hospitalizations for thyrotoxicosis (including 3,939 cases of thyroid storm), thyroid storm was independently associated with higher odds of acute heart failure (aOR: 1.15; 95% CI: 1.03–1.78), sudden cardiac death (aOR: 1.23; 95% CI: 1.04–2.17), and atrial fibrillation (aOR: 1.17; 95% CI: 1.05–2.06) compared with thyrotoxicosis without storm (3). While atrial fibrillation and high-output heart failure are the most common cardiovascular manifestations (10), malignant ventricular arrhythmias such as ventricular tachycardia and VF are exceedingly rare. In a nationwide cohort of patients hospitalized for thyroid dysfunction, Doshi *et al.* reported an incidence of VF of only 0.07% (11). In patients with thyroid storm, however, VF appears to be more frequent; a multicenter ICU study involving 92 confirmed cases found a rate of 13% (12).

Multiple factors can precipitate thyroid storm, reflecting the complex interplay between underlying hyperthyroidism and systemic stressors. Among ICU patients with thyroid storm, the most common triggers were amiodarone exposure (26%) and discontinuation of antithyroid therapy (14%), whereas one-third had no identifiable precipitant (12). Psychiatric comorbidities, particularly depression, are well recognized risk factors for medication nonadherence and may contribute to discontinuation of antithyroid therapy and progression to thyroid storm, as likely occurred in our case (13). Additional precipitants highlighted in the American Thyroid Association guidelines include infection, surgery, anesthesia, radioactive iodine therapy, myocardial infarction, diabetic ketoacidosis, and pregnancy or parturition (7).

Since the first reported case of thyroid storm presenting with ventricular tachycardia and fibrillation by Jao *et al.*, in which a woman suffered cardiac arrest and died despite prompt defibrillation and medical therapy, this presentation has been recognized as exceedingly lethal (14). Subsequent reports have reinforced this association, with cases of VF and cardiac arrest occurring in patients with uncontrolled hyperthyroidism and no structural heart disease, supporting a direct arrhythmogenic effect of thyroid hormone excess (15, 16, 17, 18). In previously reported cases, the arrhythmogenic mechanism was largely inferred rather than directly demonstrated. Our case contributes to the literature by providing direct angiographic evidence of coronary vasospasm, in the absence of obstructive coronary artery disease, as a potential trigger for malignant ventricular arrhythmia in the setting of thyroid storm.

Goldstein demonstrated that among 430 patients with angiographically documented spasm, thyrotoxicosis was present in 7.4%, often with diffuse left main coronary artery involvement and reduced responsiveness to vasodilators (19). In a case series, Choi *et al.* described eight patients with Graves’ disease who developed diffuse or severe coronary artery spasm, predominantly affecting young women (20). The proposed mechanisms include increased β-adrenergic receptor expression and sensitivity in vascular tissue (4), enhanced calcium-dependent contractility of vascular smooth muscle (5), and endothelial dysfunction. In support of the latter, Hermenegildo *et al.* found that asymmetric dimethylarginine, an endogenous inhibitor of nitric oxide synthase, is elevated in hyperthyroid patients (6).

Although the degree of coronary vasospasm observed angiographically in our case was not severe, thyroid storm is associated with increased myocardial contractility and oxygen demand (10). In this setting, such coronary vasospasm may precipitate myocardial ischemia through a supply-demand mismatch mechanism, lowering the threshold for malignant ventricular arrhythmias (21).

Thyroid hormones also exert direct electrophysiologic effects on cardiac myocytes, increasing expression and activity of sodium, potassium, and calcium channels, shortening action potential duration, increasing repolarization heterogeneity, and enhancing automaticity, all of which lower the threshold for VF (22). Additionally, hyperthyroidism induces sympathetic overactivity and reduces vagal tone, promoting QT interval variability and increasing arrhythmogenic potential (23).

Across published reports, cases of thyroid storm without structural heart disease complicated by lethal ventricular arrhythmias do not appear to share a single defining clinical profile. Restoration of euthyroidism is frequently associated with resolution of ventricular arrhythmias and prevention of recurrence (24, 25). Cases become more complex in the presence of structural heart disease, as in a report, where a patient with heart failure with reduced EF, amiodarone-induced thyroid storm, and recurrent ventricular tachycardia required venoarterial extracorporeal membrane oxygenation, catheter ablation, and ultimately total thyroidectomy to stabilize the arrhythmias (26).

The management of malignant ventricular arrhythmias in thyroid storm poses challenges. Amiodarone is frequently used in the treatment of ventricular arrhythmias but carries a substantial iodine burden, which may worsen thyrotoxicosis (27). Consequently, anti-arrhythmic strategies in this context require close collaboration between cardiology and endocrinology to balance arrhythmia control against potential thyroid-related adverse effects.

Current guidelines generally discourage ICD implantation when ventricular arrhythmias occur in the setting of a reversible cause. In the present case, VF occurred in the setting of thyroid storm, which was successfully treated with subsequent restoration of euthyroidism.

However, given the history of prior nonadherence to antithyroid therapy and the life-threatening presentation with out-of-hospital cardiac arrest, as well as concern for recurrent malignant ventricular arrhythmias, ICD implantation was pursued for secondary prevention following multidisciplinary discussion with the electrophysiology team, as an individualized clinical decision (28).

Early recognition of thyroid storm as a cause of malignant ventricular arrhythmias is essential, particularly in patients without structural heart disease with unexplained cardiac arrest. Clinicians should maintain a high index of suspicion for thyrotoxicosis in such cases, as prompt restoration of euthyroidism can be lifesaving (24, 25, 26). This case also underscores the importance of adherence to antithyroid therapy and the value of multidisciplinary management involving endocrinology, cardiology, and critical care teams. Awareness of this reversible etiology may improve outcomes through earlier diagnosis and targeted treatment.

## Declaration of interest

The authors declare that they have no conflict of interest that could be perceived as prejudicing the impartiality of the research reported.

## Funding

This research did not receive any specific grant from any funding agency in the public, commercial, or not-for-profit sector.

## Patient consent

Written informed consent for publication of their clinical details and/or clinical images was obtained from the patient.

## Author contribution statement

MHSTdeM provided direct clinical care to the patient during hospitalization and was responsible for drafting the case report, conducting the literature review, integrating clinical data, and preparing the final manuscript. MJLB contributed to the literature review and critical revision of the manuscript, ensuring accuracy and clarity of clinical and scientific content. MLMR contributed to the literature review and critical revision of the manuscript, ensuring accuracy and clarity of clinical and scientific content. EI participated in the clinical care of the patient during hospitalization; sssisted with retrieval and interpretation of coronary angiography images and contributed to the development of the clinical introduction. ED participated in the clinical care of the patient and contributed to the detailed description of the clinical presentation and hospital course. AR is the Attending physician responsible for the patient’s care who oversaw case management, supervised the clinical interpretation, and approved preparation and submission of the manuscript.
